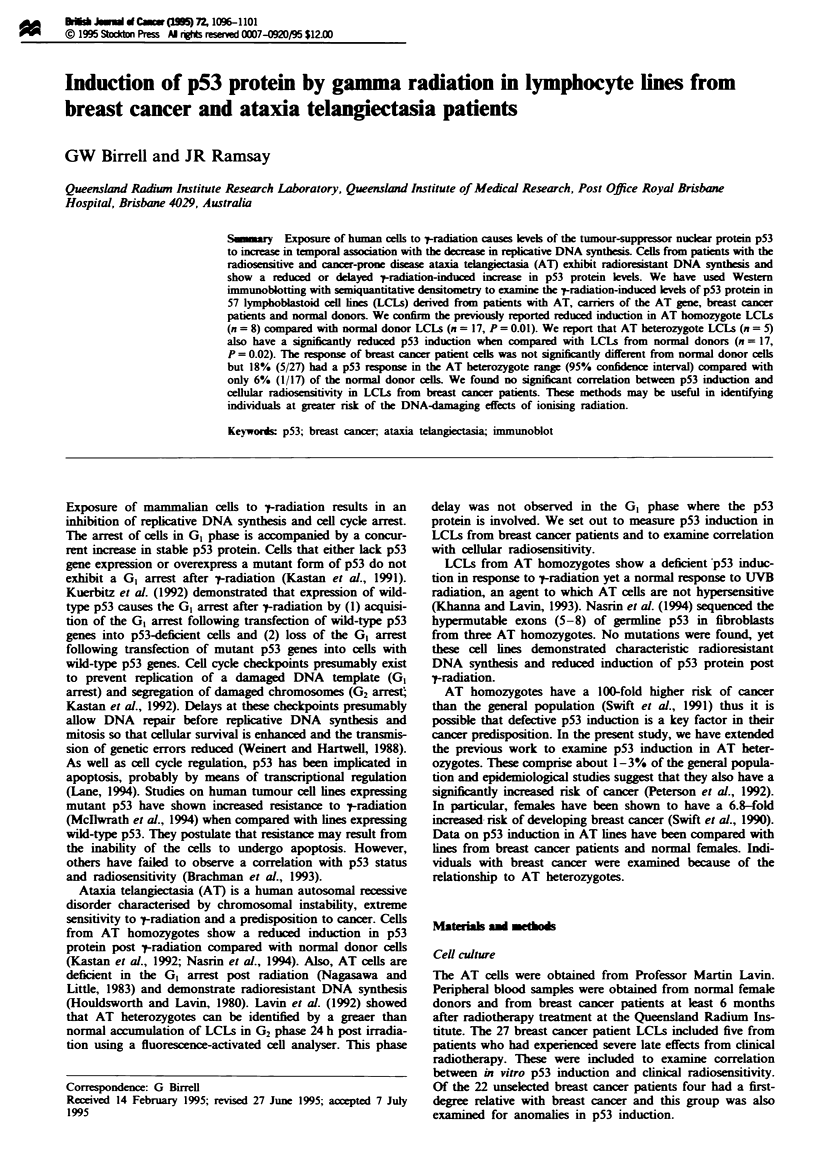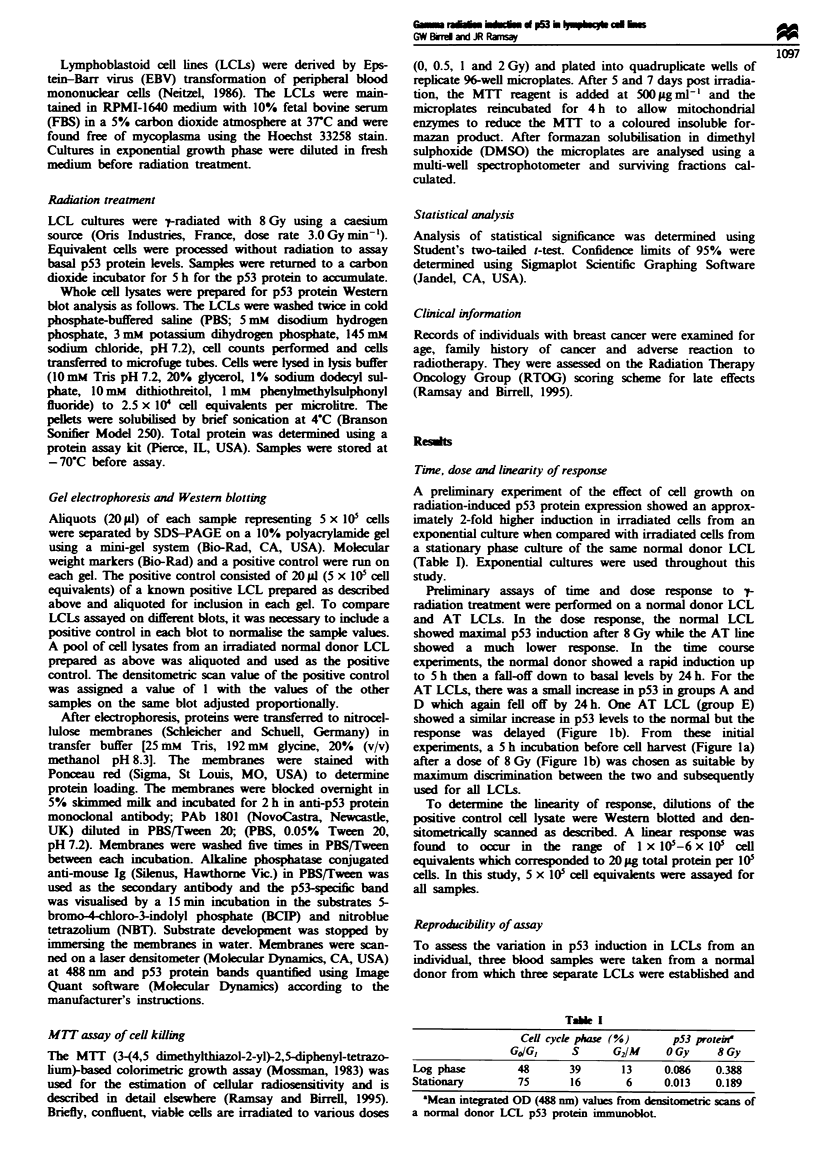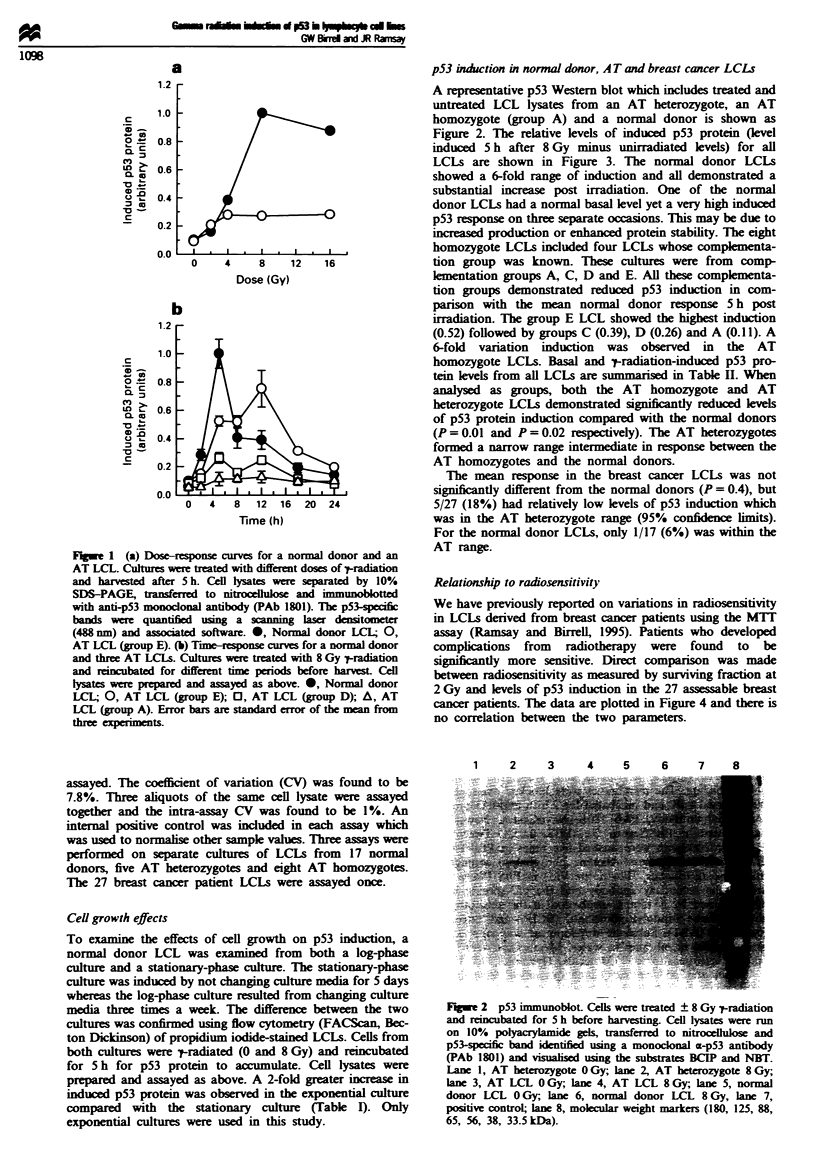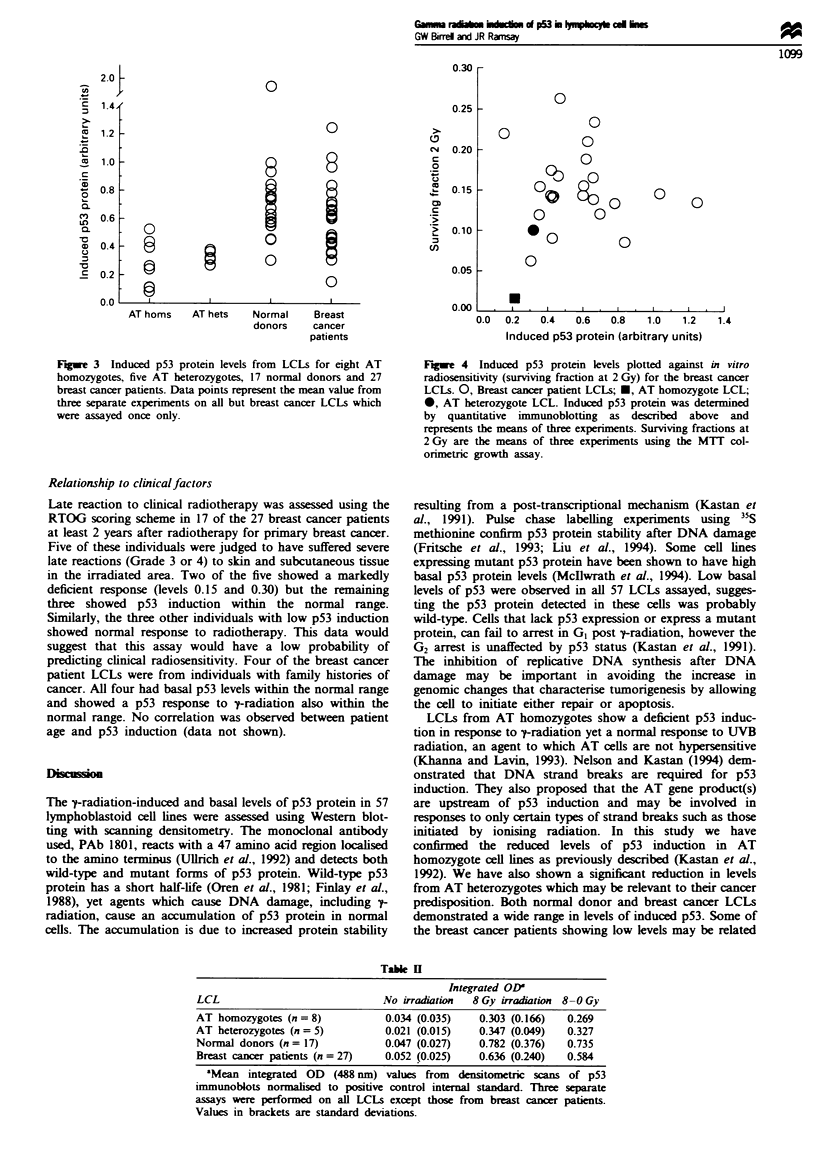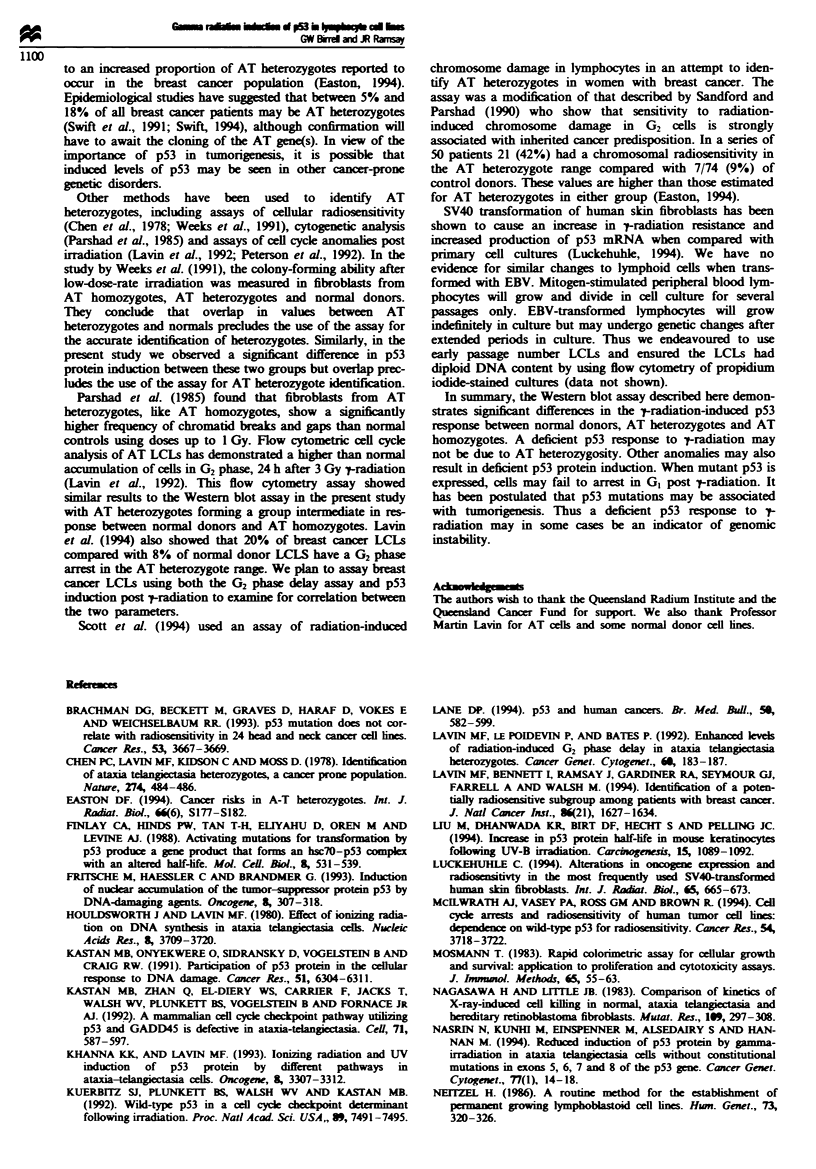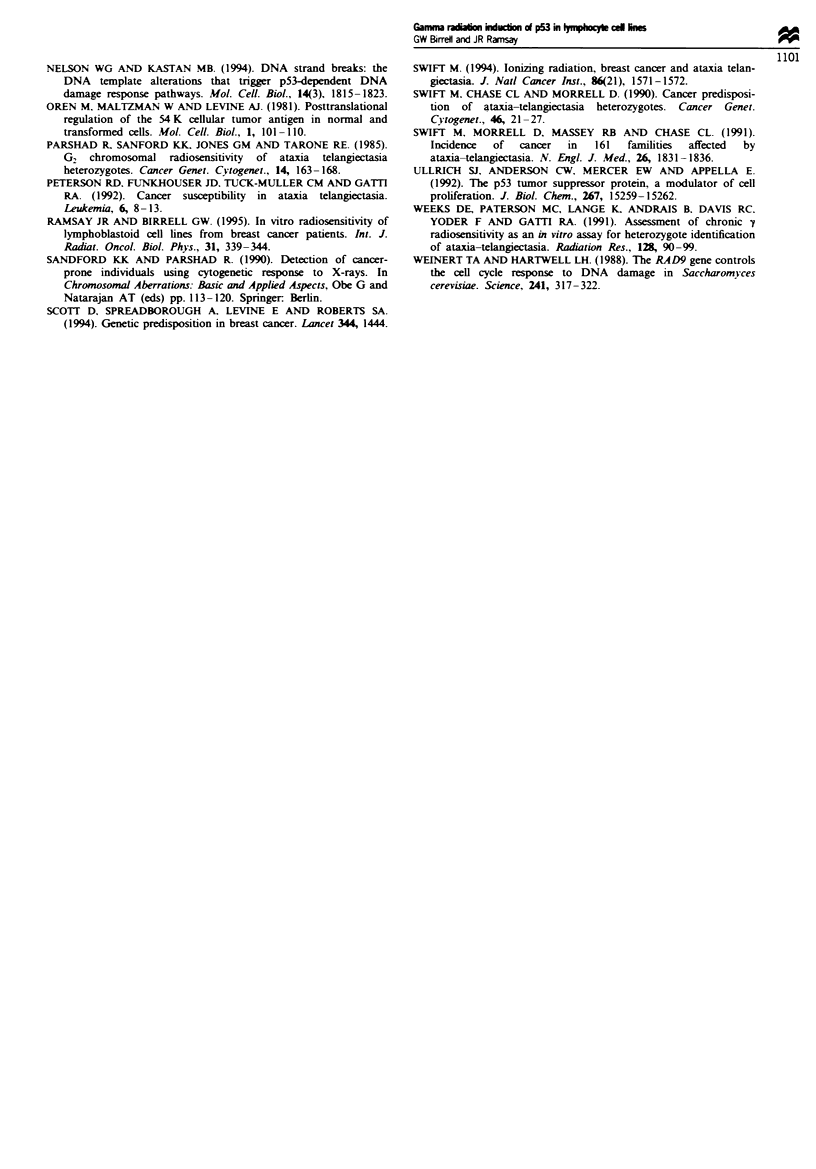# Induction of p53 protein by gamma radiation in lymphocyte lines from breast cancer and ataxia telangiectasia patients.

**DOI:** 10.1038/bjc.1995.471

**Published:** 1995-11

**Authors:** G. W. Birrell, J. R. Ramsay

**Affiliations:** Queensland Radium Institute Research Laboratory, Queensland Institute of Medical Research, Royal Brisbane Hospital, Australia.

## Abstract

**Images:**


### Induction of p53 protein by gamma radiation in lymphocyte lines from breast cancer and ataxia telangiectasia patients

GW Birrell and JR Ramsay

*Queensland Radium Institute Research Laboratory, Queensland Institute of Medical Research, Post Office Royal Brisbane Hospital, Brisbane 4029, Australia*

**Summary** Exposure of human cells to γ-radiation causes levels of the tumour-suppressor nuclear protein p53 to increase in temporal association with the decrease in replicative DNA synthesis. Cells from patients with the radiosensitive and cancer-prone disease ataxia telangiectasia (AT) exhibit radioresistant DNA synthesis and show a reduced or delayed γ-radiation-induced increase in p53 protein levels. We have used Western immunoblotting with semiquantitative densitometry to examine the γ-radiation-induced levels of p53 protein in 57 lymphoblastoid cell lines (LCLs) derived from patients with AT, carriers of the AT gene, breast cancer patients and normal donors. We confirm the previously reported reduced induction in AT homozygote LCLs ($n = 8$) compared with normal donor LCLs ($n = 17$, $P = 0.01$). We report that AT heterozygote LCLs ($n = 5$) also have a significantly reduced p53 induction when compared with LCLs from normal donors ($n = 17$, $P = 0.02$). The response of breast cancer patient cells was not significantly different from normal donor cells but 18% (5/27) had a p53 response in the AT heterozygote range (95% confidence interval) compared with only 6% (1/17) of the normal donor cells. We found no significant correlation between p53 induction and cellular radiosensitivity in LCLs from breast cancer patients. These methods may be useful in identifying individuals at greater risk of the DNA-damaging effects of ionising radiation.

**Keywords:** p53; breast cancer; ataxia telangiectasia; immunoblot

Exposure of mammalian cells to γ-radiation results in an inhibition of replicative DNA synthesis and cell cycle arrest. The arrest of cells in $G_1$ phase is accompanied by a concurrent increase in stable p53 protein. Cells that either lack p53 gene expression or overexpress a mutant form of p53 do not exhibit a $G_1$ arrest after γ-radiation (Kastan *et al.*, 1991). Kuerbitz *et al.* (1992) demonstrated that expression of wild-type p53 causes the $G_1$ arrest after γ-radiation by (1) acquisition of the $G_1$ arrest following transfection of wild-type p53 genes into p53-deficient cells and (2) loss of the $G_1$ arrest following transfection of mutant p53 genes into cells with wild-type p53 genes. Cell cycle checkpoints presumably exist to prevent replication of a damaged DNA template ($G_1$ arrest) and segregation of damaged chromosomes ($G_2$ arrest; Kastan *et al.*, 1992). Delays at these checkpoints presumably allow DNA repair before replicative DNA synthesis and mitosis so that cellular survival is enhanced and the transmission of genetic errors reduced (Weinert and Hartwell, 1988). As well as cell cycle regulation, p53 has been implicated in apoptosis, probably by means of transcriptional regulation (Lane, 1994). Studies on human tumour cell lines expressing mutant p53 have shown increased resistance to γ-radiation (McIlwrath *et al.*, 1994) when compared with lines expressing wild-type p53. They postulate that resistance may result from the inability of the cells to undergo apoptosis. However, others have failed to observe a correlation with p53 status and radiosensitivity (Brachman *et al.*, 1993).

Ataxia telangiectasia (AT) is a human autosomal recessive disorder characterised by chromosomal instability, extreme sensitivity to γ-radiation and a predisposition to cancer. Cells from AT homozygotes show a reduced induction in p53 protein post γ-radiation compared with normal donor cells (Kastan *et al.*, 1992; Nasrin *et al.*, 1994). Also, AT cells are deficient in the $G_1$ arrest post radiation (Nagasawa and Little, 1983) and demonstrate radioresistant DNA synthesis (Houldsworth and Lavin, 1980). Lavin *et al.* (1992) showed that AT heterozygotes can be identified by a greaer than normal accumulation of LCLs in $G_2$ phase 24 h post irradiation using a fluorescence-activated cell analyser. This phase delay was not observed in the $G_1$ phase where the p53 protein is involved. We set out to measure p53 induction in LCLs from breast cancer patients and to examine correlation with cellular radiosensitivity.

LCLs from AT homozygotes show a deficient p53 induction in response to γ-radiation yet a normal response to UVB radiation, an agent to which AT cells are not hypersensitive (Khanna and Lavin, 1993). Nasrin *et al.* (1994) sequenced the hypermutable exons (5–8) of germline p53 in fibroblasts from three AT homozygotes. No mutations were found, yet these cell lines demonstrated characteristic radioresistant DNA synthesis and reduced induction of p53 protein post γ-radiation.

AT homozygotes have a 100-fold higher risk of cancer than the general population (Swift *et al.*, 1991) thus it is possible that defective p53 induction is a key factor in their cancer predisposition. In the present study, we have extended the previous work to examine p53 induction in AT heterozygotes. These comprise about 1–3% of the general population and epidemiological studies suggest that they also have a significantly increased risk of cancer (Peterson *et al.*, 1992). In particular, females have been shown to have a 6.8-fold increased risk of developing breast cancer (Swift *et al.*, 1990). Data on p53 induction in AT lines have been compared with lines from breast cancer patients and normal females. Individuals with breast cancer were examined because of the relationship to AT heterozygotes.

### Materials and methods

### Cell culture

The AT cells were obtained from Professor Martin Lavin. Peripheral blood samples were obtained from normal female donors and from breast cancer patients at least 6 months after radiotherapy treatment at the Queensland Radium Institute. The 27 breast cancer patient LCLs included five from patients who had experienced severe late effects from clinical radiotherapy. These were included to examine correlation between *in vitro* p53 induction and clinical radiosensitivity. Of the 22 unselected breast cancer patients four had a first-degree relative with breast cancer and this group was also examined for anomalies in p53 induction.

Correspondence: G Birrell
Received 14 February 1995; revised 27 June 1995; accepted 7 July 1995

Lymphoblastoid cell lines (LCLs) were derived by Epstein–Barr virus (EBV) transformation of peripheral blood mononuclear cells (Neitzel, 1986). The LCLs were maintained in RPMI-1640 medium with 10% fetal bovine serum (FBS) in a 5% carbon dioxide atmosphere at 37°C and were found free of mycoplasma using the Hoechst 33258 stain. Cultures in exponential growth phase were diluted in fresh medium before radiation treatment.

*Radiation treatment*

LCL cultures were γ-radiated with 8 Gy using a caesium source (Oris Industries, France, dose rate 3.0 Gy $min^{-1}$). Equivalent cells were processed without radiation to assay basal p53 protein levels. Samples were returned to a carbon dioxide incubator for 5 h for the p53 protein to accumulate.

Whole cell lysates were prepared for p53 protein Western blot analysis as follows. The LCLs were washed twice in cold phosphate-buffered saline (PBS; 5 mM disodium hydrogen phosphate, 3 mM potassium dihydrogen phosphate, 145 mM sodium chloride, pH 7.2), cell counts performed and cells transferred to microfuge tubes. Cells were lysed in lysis buffer (10 mM Tris pH 7.2, 20% glycerol, 1% sodium dodecyl sulphate, 10 mM dithiothreitol, 1 mM phenylmethylsulphonyl fluoride) to $2.5 \times 10^4$ cell equivalents per microlitre. The pellets were solubilised by brief sonication at 4°C (Branson Sonifier Model 250). Total protein was determined using a protein assay kit (Pierce, IL, USA). Samples were stored at −70°C before assay.

*Gel electrophoresis and Western blotting*

Aliquots (20 µl) of each sample representing $5 \times 10^5$ cells were separated by SDS–PAGE on a 10% polyacrylamide gel using a mini-gel system (Bio-Rad, CA, USA). Molecular weight markers (Bio-Rad) and a positive control were run on each gel. The positive control consisted of 20 µl ($5 \times 10^5$ cell equivalents) of a known positive LCL prepared as described above and aliquoted for inclusion in each gel. To compare LCLs assayed on different blots, it was necessary to include a positive control in each blot to normalise the sample values. A pool of cell lysates from an irradiated normal donor LCL prepared as above was aliquoted and used as the positive control. The densitometric scan value of the positive control was assigned a value of 1 with the values of the other samples on the same blot adjusted proportionally.

After electrophoresis, proteins were transferred to nitrocellulose membranes (Schleicher and Schuell, Germany) in transfer buffer [25 mM Tris, 192 mM glycine, 20% (v/v) methanol pH 8.3]. The membranes were stained with Ponceau red (Sigma, St Louis, MO, USA) to determine protein loading. The membranes were blocked overnight in 5% skimmed milk and incubated for 2 h in anti-p53 protein monoclonal antibody; PAb 1801 (NovoCastra, Newcastle, UK) diluted in PBS/Tween 20; (PBS, 0.05% Tween 20, pH 7.2). Membranes were washed five times in PBS/Tween between each incubation. Alkaline phosphatase conjugated anti-mouse Ig (Silenus, Hawthorne Vic.) in PBS/Tween was used as the secondary antibody and the p53-specific band was visualised by a 15 min incubation in the substrates 5-bromo-4-chloro-3-indolyl phosphate (BCIP) and nitroblue tetrazolium (NBT). Substrate development was stopped by immersing the membranes in water. Membranes were scanned on a laser densitometer (Molecular Dynamics, CA, USA) at 488 nm and p53 protein bands quantified using Image Quant software (Molecular Dynamics) according to the manufacturer's instructions.

*MTT assay of cell killing*

The MTT (3-(4,5 dimethylthiazol-2-yl)-2,5-diphenyl-tetrazolium)-based colorimetric growth assay (Mossman, 1983) was used for the estimation of cellular radiosensitivity and is described in detail elsewhere (Ramsay and Birrell, 1995). Briefly, confluent, viable cells are irradiated to various doses (0, 0.5, 1 and 2 Gy) and plated into quadruplicate wells of replicate 96-well microplates. After 5 and 7 days post irradiation, the MTT reagent is added at 500 µg $ml^{-1}$ and the microplates reincubated for 4 h to allow mitochondrial enzymes to reduce the MTT to a coloured insoluble formazan product. After formazan solubilisation in dimethyl sulphoxide (DMSO) the microplates are analysed using a multi-well spectrophotometer and surviving fractions calculated.

*Statistical analysis*

Analysis of statistical significance was determined using Student's two-tailed *t*-test. Confidence limits of 95% were determined using Sigmaplot Scientific Graphing Software (Jandel, CA, USA).

*Clinical information*

Records of individuals with breast cancer were examined for age, family history of cancer and adverse reaction to radiotherapy. They were assessed on the Radiation Therapy Oncology Group (RTOG) scoring scheme for late effects (Ramsay and Birrell, 1995).

### Results

*Time, dose and linearity of response*

A preliminary experiment of the effect of cell growth on radiation-induced p53 protein expression showed an approximately 2-fold higher induction in irradiated cells from an exponential culture when compared with irradiated cells from a stationary phase culture of the same normal donor LCL (Table I). Exponential cultures were used throughout this study.

Preliminary assays of time and dose response to γ-radiation treatment were performed on a normal donor LCL and AT LCLs. In the dose response, the normal LCL showed maximal p53 induction after 8 Gy while the AT line showed a much lower response. In the time course experiments, the normal donor showed a rapid induction up to 5 h then a fall-off down to basal levels by 24 h. For the AT LCLs, there was a small increase in p53 in groups A and D which again fell off by 24 h. One AT LCL (group E) showed a similar increase in p53 levels to the normal but the response was delayed (Figure 1b). From these initial experiments, a 5 h incubation before cell harvest (Figure 1a) after a dose of 8 Gy (Figure 1b) was chosen as suitable by maximum discrimination between the two and subsequently used for all LCLs.

To determine the linearity of response, dilutions of the positive control cell lysate were Western blotted and densitometrically scanned as described. A linear response was found to occur in the range of $1 \times 10^5$–$6 \times 10^5$ cell equivalents which corresponded to 20 µg total protein per $10^5$ cells. In this study, $5 \times 10^5$ cell equivalents were assayed for all samples.

*Reproducibility of assay*

To assess the variation in p53 induction in LCLs from an individual, three blood samples were taken from a normal donor from which three separate LCLs were established and

**Table I**

| | Cell cycle phase (%) | | | p53 protein[a] | |
|---|---|---|---|---|---|
| | $G_0/G_1$ | S | $G_2/M$ | 0 Gy | 8 Gy |
| Log phase | 48 | 39 | 13 | 0.086 | 0.388 |
| Stationary | 75 | 16 | 6 | 0.013 | 0.189 |

[a]Mean integrated OD (488 nm) values from densitometric scans of a normal donor LCL p53 protein immunoblot.

**Figure 1** (a) Dose–response curves for a normal donor and an AT LCL. Cultures were treated with different doses of γ-radiation and harvested after 5 h. Cell lysates were separated by 10% SDS–PAGE, transferred to nitrocellulose and immunoblotted with anti-p53 monoclonal antibody (PAb 1801). The p53-specific bands were quantified using a scanning laser densitometer (488 nm) and associated software. ●, Normal donor LCL; ○, AT LCL (group E). (b) Time–response curves for a normal donor and three AT LCLs. Cultures were treated with 8 Gy γ-radiation and reincubated for different time periods before harvest. Cell lysates were prepared and assayed as above. ●, Normal donor LCL; ○, AT LCL (group E); □, AT LCL (group D); Δ, AT LCL (group A). Error bars are standard error of the mean from three experiments.

assayed. The coefficient of variation (CV) was found to be 7.8%. Three aliquots of the same cell lysate were assayed together and the intra-assay CV was found to be 1%. An internal positive control was included in each assay which was used to normalise other sample values. Three assays were performed on separate cultures of LCLs from 17 normal donors, five AT heterozygotes and eight AT homozygotes. The 27 breast cancer patient LCLs were assayed once.

### *Cell growth effects*

To examine the effects of cell growth on p53 induction, a normal donor LCL was examined from both a log-phase culture and a stationary-phase culture. The stationary-phase culture was induced by not changing culture media for 5 days whereas the log-phase culture resulted from changing culture media three times a week. The difference between the two cultures was confirmed using flow cytometry (FACScan, Becton Dickinson) of propidium iodide-stained LCLs. Cells from both cultures were γ-radiated (0 and 8 Gy) and reincubated for 5 h for p53 protein to accumulate. Cell lysates were prepared and assayed as above. A 2-fold greater increase in induced p53 protein was observed in the exponential culture compared with the stationary culture (Table I). Only exponential cultures were used in this study.

### *p53 induction in normal donor, AT and breast cancer LCLs*

A representative p53 Western blot which includes treated and untreated LCL lysates from an AT heterozygote, an AT homozygote (group A) and a normal donor is shown as Figure 2. The relative levels of induced p53 protein (level induced 5 h after 8 Gy minus unirradiated levels) for all LCLs are shown in Figure 3. The normal donor LCLs showed a 6-fold range of induction and all demonstrated a substantial increase post irradiation. One of the normal donor LCLs had a normal basal level yet a very high induced p53 response on three separate occasions. This may be due to increased production or enhanced protein stability. The eight homozygote LCLs included four LCLs whose complementation group was known. These cultures were from complementation groups A, C, D and E. All these complementation groups demonstrated reduced p53 induction in comparison with the mean normal donor response 5 h post irradiation. The group E LCL showed the highest induction (0.52) followed by groups C (0.39), D (0.26) and A (0.11). A 6-fold variation induction was observed in the AT homozygote LCLs. Basal and γ-radiation-induced p53 protein levels from all LCLs are summarised in Table II. When analysed as groups, both the AT homozygote and AT heterozygote LCLs demonstrated significantly reduced levels of p53 protein induction compared with the normal donors ($P = 0.01$ and $P = 0.02$ respectively). The AT heterozygotes formed a narrow range intermediate in response between the AT homozygotes and the normal donors.

The mean response in the breast cancer LCLs was not significantly different from the normal donors ($P = 0.4$), but 5/27 (18%) had relatively low levels of p53 induction which was in the AT heterozygote range (95% confidence limits). For the normal donor LCLs, only 1/17 (6%) was within the AT range.

### *Relationship to radiosensitivity*

We have previously reported on variations in radiosensitivity in LCLs derived from breast cancer patients using the MTT assay (Ramsay and Birrell, 1995). Patients who developed complications from radiotherapy were found to be significantly more sensitive. Direct comparison was made between radiosensitivity as measured by surviving fraction at 2 Gy and levels of p53 induction in the 27 assessable breast cancer patients. The data are plotted in Figure 4 and there is no correlation between the two parameters.

**Figure 2** p53 immunoblot. Cells were treated ± 8 Gy γ-radiation and reincubated for 5 h before harvesting. Cell lysates were run on 10% polyacrylamide gels, transferred to nitrocellulose and p53-specific band identified using a monoclonal α-p53 antibody (PAb 1801) and visualised using the substrates BCIP and NBT. Lane 1, AT heterozygote 0 Gy; lane 2, AT heterozygote 8 Gy; lane 3, AT LCL 0 Gy; lane 4, AT LCL 8 Gy; lane 5, normal donor LCL 0 Gy; lane 6, normal donor LCL 8 Gy, lane 7, positive control; lane 8, molecular weight markers (180, 125, 88, 65, 56, 38, 33.5 kDa).

**Figure 3** Induced p53 protein levels from LCLs for eight AT homozygotes, five AT heterozygotes, 17 normal donors and 27 breast cancer patients. Data points represent the mean value from three separate experiments on all but breast cancer LCLs which were assayed once only.

**Figure 4** Induced p53 protein levels plotted against *in vitro* radiosensitivity (surviving fraction at 2 Gy) for the breast cancer LCLs. ○, Breast cancer patient LCLs; ■, AT homozygote LCL; ●, AT heterozygote LCL. Induced p53 protein was determined by quantitative immunoblotting as described above and represents the means of three experiments. Surviving fractions at 2 Gy are the means of three experiments using the MTT colorimetric growth assay.

*Relationship to clinical factors*

Late reaction to clinical radiotherapy was assessed using the RTOG scoring scheme in 17 of the 27 breast cancer patients at least 2 years after radiotherapy for primary breast cancer. Five of these individuals were judged to have suffered severe late reactions (Grade 3 or 4) to skin and subcutaneous tissue in the irradiated area. Two of the five showed a markedly deficient response (levels 0.15 and 0.30) but the remaining three showed p53 induction within the normal range. Similarly, the three other individuals with low p53 induction showed normal response to radiotherapy. This data would suggest that this assay would have a low probability of predicting clinical radiosensitivity. Four of the breast cancer patient LCLs were from individuals with family histories of cancer. All four had basal p53 levels within the normal range and showed a p53 response to γ-radiation also within the normal range. No correlation was observed between patient age and p53 induction (data not shown).

### Discussion

The γ-radiation-induced and basal levels of p53 protein in 57 lymphoblastoid cell lines were assessed using Western blotting with scanning densitometry. The monoclonal antibody used, PAb 1801, reacts with a 47 amino acid region localised to the amino terminus (Ullrich *et al.*, 1992) and detects both wild-type and mutant forms of p53 protein. Wild-type p53 protein has a short half-life (Oren *et al.*, 1981; Finlay *et al.*, 1988), yet agents which cause DNA damage, including γ-radiation, cause an accumulation of p53 protein in normal cells. The accumulation is due to increased protein stability resulting from a post-transcriptional mechanism (Kastan *et al.*, 1991). Pulse chase labelling experiments using $^{35}S$ methionine confirm p53 protein stability after DNA damage (Fritsche *et al.*, 1993; Liu *et al.*, 1994). Some cell lines expressing mutant p53 protein have been shown to have high basal p53 protein levels (McIlwrath *et al.*, 1994). Low basal levels of p53 were observed in all 57 LCLs assayed, suggesting the p53 protein detected in these cells was probably wild-type. Cells that lack p53 expression or express a mutant protein, can fail to arrest in $G_1$ post γ-radiation, however the $G_2$ arrest is unaffected by p53 status (Kastan *et al.*, 1991). The inhibition of replicative DNA synthesis after DNA damage may be important in avoiding the increase in genomic changes that characterise tumorigenesis by allowing the cell to initiate either repair or apoptosis.

LCLs from AT homozygotes show a deficient p53 induction in response to γ-radiation yet a normal response to UVB radiation, an agent to which AT cells are not hypersensitive (Khanna and Lavin, 1993). Nelson and Kastan (1994) demonstrated that DNA strand breaks are required for p53 induction. They also proposed that the AT gene product(s) are upstream of p53 induction and may be involved in responses to only certain types of strand breaks such as those initiated by ionising radiation. In this study we have confirmed the reduced levels of p53 induction in AT homozygote cell lines as previously described (Kastan *et al.*, 1992). We have also shown a significant reduction in levels from AT heterozygotes which may be relevant to their cancer predisposition. Both normal donor and breast cancer LCLs demonstrated a wide range in levels of induced p53. Some of the breast cancer patients showing low levels may be related

**Table II**

| *LCL* | *Integrated OD*[a] *No irradiation* | *8 Gy irradiation* | *8–0 Gy* |
|---|---|---|---|
| AT homozygotes ($n = 8$) | 0.034 (0.035) | 0.303 (0.166) | 0.269 |
| AT heterozygotes ($n = 5$) | 0.021 (0.015) | 0.347 (0.049) | 0.327 |
| Normal donors ($n = 17$) | 0.047 (0.027) | 0.782 (0.376) | 0.735 |
| Breast cancer patients ($n = 27$) | 0.052 (0.025) | 0.636 (0.240) | 0.584 |

[a]Mean integrated OD (488 nm) values from densitometric scans of p53 immunoblots normalised to positive control internal standard. Three separate assays were performed on all LCLs except those from breast cancer patients. Values in brackets are standard deviations.

to an increased proportion of AT heterozygotes reported to occur in the breast cancer population (Easton, 1994). Epidemiological studies have suggested that between 5% and 18% of all breast cancer patients may be AT heterozygotes (Swift *et al.*, 1991; Swift, 1994), although confirmation will have to await the cloning of the AT gene(s). In view of the importance of p53 in tumorigenesis, it is possible that induced levels of p53 may be seen in other cancer-prone genetic disorders.

Other methods have been used to identify AT heterozygotes, including assays of cellular radiosensitivity (Chen *et al.*, 1978; Weeks *et al.*, 1991), cytogenetic analysis (Parshad *et al.*, 1985) and assays of cell cycle anomalies post irradiation (Lavin *et al.*, 1992; Peterson *et al.*, 1992). In the study by Weeks *et al.* (1991), the colony-forming ability after low-dose-rate irradiation was measured in fibroblasts from AT homozygotes, AT heterozygotes and normal donors. They conclude that overlap in values between AT heterozygotes and normals precludes the use of the assay for the accurate identification of heterozygotes. Similarly, in the present study we observed a significant difference in p53 protein induction between these two groups but overlap precludes the use of the assay for AT heterozygote identification.

Parshad *et al.* (1985) found that fibroblasts from AT heterozygotes, like AT homozygotes, show a significantly higher frequency of chromatid breaks and gaps than normal controls using doses up to 1 Gy. Flow cytometric cell cycle analysis of AT LCLs has demonstrated a higher than normal accumulation of cells in $G_2$ phase, 24 h after 3 Gy $\gamma$-radiation (Lavin *et al.*, 1992). This flow cytometry assay showed similar results to the Western blot assay in the present study with AT heterozygotes forming a group intermediate in response between normal donors and AT homozygotes. Lavin *et al.* (1994) also showed that 20% of breast cancer LCLs compared with 8% of normal donor LCLS have a $G_2$ phase arrest in the AT heterozygote range. We plan to assay breast cancer LCLs using both the $G_2$ phase delay assay and p53 induction post $\gamma$-radiation to examine for correlation between the two parameters.

Scott *et al.* (1994) used an assay of radiation-induced chromosome damage in lymphocytes in an attempt to identify AT heterozygotes in women with breast cancer. The assay was a modification of that described by Sandford and Parshad (1990) who show that sensitivity to radiation-induced chromosome damage in $G_2$ cells is strongly associated with inherited cancer predisposition. In a series of 50 patients 21 (42%) had a chromosomal radiosensitivity in the AT heterozygote range compared with 7/74 (9%) of control donors. These values are higher than those estimated for AT heterozygotes in either group (Easton, 1994).

SV40 transformation of human skin fibroblasts has been shown to cause an increase in $\gamma$-radiation resistance and increased production of p53 mRNA when compared with primary cell cultures (Luckehuhle, 1994). We have no evidence for similar changes to lymphoid cells when transformed with EBV. Mitogen-stimulated peripheral blood lymphocytes will grow and divide in cell culture for several passages only. EBV-transformed lymphocytes will grow indefinitely in culture but may undergo genetic changes after extended periods in culture. Thus we endeavoured to use early passage number LCLs and ensured the LCLs had diploid DNA content by using flow cytometry of propidium iodide-stained cultures (data not shown).

In summary, the Western blot assay described here demonstrates significant differences in the $\gamma$-radiation-induced p53 response between normal donors, AT heterozygotes and AT homozygotes. A deficient p53 response to $\gamma$-radiation may not be due to AT heterozygosity. Other anomalies may also result in deficient p53 protein induction. When mutant p53 is expressed, cells may fail to arrest in $G_1$ post $\gamma$-radiation. It has been postulated that p53 mutations may be associated with tumorigenesis. Thus a deficient p53 response to $\gamma$-radiation may in some cases be an indicator of genomic instability.

**Acknowledgements**
The authors wish to thank the Queensland Radium Institute and the Queensland Cancer Fund for support. We also thank Professor Martin Lavin for AT cells and some normal donor cell lines.

**References**

BRACHMAN DG, BECKETT M, GRAVES D, HARAF D, VOKES E AND WEICHSELBAUM RR. (1993). p53 mutation does not correlate with radiosensitivity in 24 head and neck cancer cell lines. *Cancer Res.*, **53**, 3667–3669.

CHEN PC, LAVIN MF, KIDSON C AND MOSS D. (1978). Identification of ataxia telangiectasia heterozygotes, a cancer prone population. *Nature*, **274**, 484–486.

EASTON DF. (1994). Cancer risks in A-T heterozygotes. *Int. J. Radiat. Biol.*, **66**(6), S177–S182.

FINLAY CA, HINDS PW, TAN T-H, ELIYAHU D, OREN M AND LEVINE AJ. (1988). Activating mutations for transformation by p53 produce a gene product that forms an hsc70-p53 complex with an altered half-life. *Mol. Cell. Biol.*, **8**, 531–539.

FRITSCHE M, HAESSLER C AND BRANDMER G. (1993). Induction of nuclear accumulation of the tumor-suppressor protein p53 by DNA-damaging agents. *Oncogene*, **8**, 307–318.

HOULDSWORTH J AND LAVIN MF. (1980). Effect of ionizing radiation on DNA synthesis in ataxia telangiectasia cells. *Nucleic Acids Res.*, **8**, 3709–3720.

KASTAN MB, ONYEKWERE O, SIDRANSKY D, VOGELSTEIN B AND CRAIG RW. (1991). Participation of p53 protein in the cellular response to DNA damage. *Cancer Res.*, **51**, 6304–6311.

KASTAN MB, ZHAN Q, EL-DIERY WS, CARRIER F, JACKS T, WALSH WV, PLUNKETT BS, VOGELSTEIN B AND FORNACE JR AJ. (1992). A mammalian cell cycle checkpoint pathway utilizing p53 and GADD45 is defective in ataxia-telangiectasia. *Cell*, **71**, 587–597.

KHANNA KK, AND LAVIN MF. (1993). Ionizing radiation and UV induction of p53 protein by different pathways in ataxia-telangiectasia cells. *Oncogene*, **8**, 3307–3312.

KUERBITZ SJ, PLUNKETT BS, WALSH WV AND KASTAN MB. (1992). Wild-type p53 in a cell cycle checkpoint determinant following irradiation. *Proc. Natl Acad. Sci. USA*,, **89**, 7491–7495.

LANE DP. (1994). p53 and human cancers. *Br. Med. Bull.*, **50**, 582–599.

LAVIN MF, LE POIDEVIN P, AND BATES P. (1992). Enhanced levels of radiation-induced $G_2$ phase delay in ataxia telangiectasia heterozygotes. *Cancer Genet. Cytogenet.*, **60**, 183–187.

LAVIN MF, BENNETT I, RAMSAY J, GARDINER RA, SEYMOUR GJ, FARRELL A AND WALSH M. (1994). Identification of a potentially radiosensitive subgroup among patients with breast cancer. *J. Natl Cancer Inst.*, **86**(21), 1627–1634.

LIU M, DHANWADA KR, BIRT DF, HECHT S AND PELLING JC. (1994). Increase in p53 protein half-life in mouse keratinocytes following UV-B irradiation. *Carcinogenesis*, **15**, 1089–1092.

LUCKEHUHLE C. (1994). Alterations in oncogene expression and radiosensitivty in the most frequently used SV40-transformed human skin fibroblasts. *Int. J. Radiat. Biol.*, **65**, 665–673.

MCILWRATH AJ, VASEY PA, ROSS GM AND BROWN R. (1994). Cell cycle arrests and radiosensitivity of human tumor cell lines: dependence on wild-type p53 for radiosensitivity. *Cancer Res.*, **54**, 3718–3722.

MOSMANN T. (1983). Rapid colorimetric assay for cellular growth and survival: application to proliferation and cytotoxicity assays. *J. Immunol. Methods*, **65**, 55–63.

NAGASAWA H AND LITTLE JB. (1983). Comparison of kinetics of X-ray-induced cell killing in normal, ataxia telangiectasia and hereditary retinoblastoma fibroblasts. *Mutat. Res.*, **109**, 297–308.

NASRIN N, KUNHI M, EINSPENNER M, ALSEDAIRY S AND HANNAN M. (1994). Reduced induction of p53 protein by gamma-irradiation in ataxia telangiectasia cells without constitutional mutations in exons 5, 6, 7 and 8 of the p53 gene. *Cancer Genet. Cytogenet.*, **77**(1), 14–18.

NEITZEL H. (1986). A routine method for the establishment of permanent growing lymphoblastoid cell lines. *Hum. Genet.*, **73**, 320–326.

NELSON WG AND KASTAN MB. (1994). DNA strand breaks: the DNA template alterations that trigger p53-dependent DNA damage response pathways. *Mol. Cell. Biol.*, **14**(3), 1815–1823.
OREN M, MALTZMAN W AND LEVINE AJ. (1981). Posttranslational regulation of the 54 K cellular tumor antigen in normal and transformed cells. *Mol. Cell. Biol.*, **1**, 101–110.
PARSHAD R, SANFORD KK, JONES GM AND TARONE RE. (1985). $G_2$ chromosomal radiosensitivity of ataxia telangiectasia heterozygotes. *Cancer Genet. Cytogenet.*, **14**, 163–168.
PETERSON RD, FUNKHOUSER JD, TUCK-MULLER CM AND GATTI RA. (1992). Cancer susceptibility in ataxia telangiectasia. *Leukemia*, **6**, 8–13.
RAMSAY JR AND BIRRELL GW. (1995). In vitro radiosensitivity of lymphoblastoid cell lines from breast cancer patients. *Int. J. Radiat. Oncol. Biol. Phys.*, **31**, 339–344.
SANDFORD KK AND PARSHAD R. (1990). Detection of cancer-prone individuals using cytogenetic response to X-rays. In *Chromosomal Aberrations: Basic and Applied Aspects*, Obe G and Natarajan AT (eds) pp. 113–120. Springer: Berlin.
SCOTT D, SPREADBOROUGH A, LEVINE E AND ROBERTS SA. (1994). Genetic predisposition in breast cancer. *Lancet* **344**, 1444.
SWIFT M. (1994). Ionizing radiation, breast cancer and ataxia telangiectasia. *J. Natl Cancer Inst.*, **86**(21), 1571–1572.
SWIFT M, CHASE CL AND MORRELL D. (1990). Cancer predisposition of ataxia–telangiectasia heterozygotes. *Cancer Genet. Cytogenet.*, **46**, 21–27.
SWIFT M, MORRELL D, MASSEY RB AND CHASE CL. (1991). Incidence of cancer in 161 families affected by ataxia–telangiectasia. *N. Engl. J. Med.*, **26**, 1831–1836.
ULLRICH SJ, ANDERSON CW, MERCER EW AND APPELLA E. (1992). The p53 tumor suppressor protein, a modulator of cell proliferation. *J. Biol. Chem.*, **267**, 15259–15262.
WEEKS DE, PATERSON MC, LANGE K, ANDRAIS B, DAVIS RC, YODER F AND GATTI RA. (1991). Assessment of chronic γ radiosensitivity as an *in vitro* assay for heterozygote identification of ataxia–telangiectasia. *Radiation Res.*, **128**, 90–99.
WEINERT TA AND HARTWELL LH. (1988). The *RAD9* gene controls the cell cycle response to DNA damage in *Saccharomyces cerevisiae*. *Science*, **241**, 317–322.